# Headache in long COVID as disabling condition: A clinical approach

**DOI:** 10.3389/fneur.2023.1149294

**Published:** 2023-03-23

**Authors:** Arthur Nascimento Rodrigues, Apio Ricardo Nazareth Dias, Alna Carolina Mendes Paranhos, Camilla Costa Silva, Thalita da Rocha Bastos, Bárbara Barros de Brito, Nívia Monteiro da Silva, Emanuel de Jesus Soares de Sousa, Juarez Antônio Simões Quaresma, Luiz Fábio Magno Falcão

**Affiliations:** ^1^Center for Biological Health Sciences, State University of Pará, Belém, Pará, Brazil; ^2^Tropical Medicine Center, Federal University of Pará, Belém, Pará, Brazil; ^3^USP Medical School, São Paulo University, São Paulo, São Paulo, Brazil

**Keywords:** coronavirus infection, long COVID-19 syndrome, post-COVID-19, headache, head pain

## Abstract

**Background and purpose:**

Severe acute respiratory syndrome coronavirus 2 (SARS-CoV-2) infection can exacerbate previous headache disorders or change the type of pain experienced from headaches. This study aimed to investigate the clinical features of Long COVID headaches.

**Method:**

This was a cross-sectional, descriptive, and analytical observational study that included 102 patients (with previous headache, *n* = 50; without previous headache, *n* = 52) with long COVID and headache complaints. The Migraine Disability Assessment Test and Visual Analog Pain Scale were used to collect participants' headache data according to a standardized protocol.

**Results:**

The patients in this study who reported experiencing headaches before COVID-19 had longer headache duration in the long COVID phase than that in the pre-long COVID phase (*p* = 0.031), exhibited partial improvement in headache symptoms with analgesics (*p* = 0.045), and had a duration of long COVID of <1 year (*p* = 0.030). Patients with moderate or severe disability and those classified as having severe headaches in the long COVID phase were highly likely to develop chronic headaches. Hospital admission [odds ratio (OR) = 3.0082; 95% confidence interval (95% CI): 1.10–8.26], back pain (OR = 4.0017; 95% CI: 1.13–14.17), insomnia (OR = 3.1339; 95% CI: 1.39–7.06), and paraesthesia (OR = 2.7600; 95% CI: 1.20–6.33) were associated with headache in these patients.

**Conclusion:**

Headache is a disabling condition in patients with long COVID-19, exacerbating the conditions of those with headaches prior to contracting COVID-19.

## 1. Introduction

After the acute period of coronavirus disease 2019 (COVID-19), some symptoms persist for months, or new symptoms appear, such as muscle fatigue, arthralgia, myalgia, cognitive decline, and anxiety, among others (1). This stage is called long COVID syndrome (hereafter referred to as “long COVID”) (2, 3) and includes the following diagnostic criteria: patients with a previous diagnosis of COVID-19, presentation of symptoms at least 4 weeks after the acute phase of the disease, and symptoms that are present for at least 3 months and that appear no later than 2 months after infection (4). The major late neurological manifestations include hyposmia, hypogeusia, dizziness, insomnia, and headache characterized by intense and frequent chronic pain that is resistant to easily accessible drugs (3, 5–7).

To diagnose chronic headache attributed to systemic viral infection (8), the International Classification of Systemic Headaches (ICHD-3) uses a duration >3 months after the acute phase of a viral disease. According to the ICHD-3, chronic daily headache is defined as 15 or more headache attacks per month for at least 3 months (8), and these can be associated with chronic fatigue, anxiety, depression, and sleep disorders (9). The clinical presentation of headache in long COVID is similar to that of new daily persistent headache (NDPH), as these patients have daily pain and low response to common treatment. Although patients can have different migraine phenotypes, the tension-type headache phenotype is the most common in patients with long COVID due to its bilateral and pressing quality characteristics (6, 7, 10). Long COVID headache can last for more than 6 months after the acute stage of the disease, and many of these patients may not have experienced headache prior to contracting COVID-19 (3). Thus, COVID-19 infection may be associated with new onset of headaches or worsening of pre-existing headache conditions (3).

The pathophysiology of long COVID headache is still not completely known. However, it has been hypothesized that the direct invasion of severe acute respiratory syndrome coronavirus 2 (SARS-CoV-2) into the central nervous system affects neuronal pathways (3). For example, a prospective study reported a reduction in gray matter in the parahippocampal gyrus and orbitofrontal cortex in patients with COVID-19 (11). In addition, headache associated with viral disease activates the immune system functions, as patients with Long COVID headache demonstrate increased blood levels of cytokines and interleukins, which can indicate the persistent activation of the immune system (10, 12–14).

The exact pathophysiological mechanism underlying headaches associated with long COVID, as well as the clinical features in these patients, represents a gap in scientific knowledge that remains to be filled. Thus, this study aimed to assess the clinical characteristics of long COVID headache, including its intensity and effect on disability, as well as its associated epidemiological factors.

## 2. Methods

### 2.1. Ethical aspects

This study was approved by the Ethics Committee and for Research with Human Beings at the Center for Biological Health Sciences of the University of the State of Pará (opinion 3,619,141). All participants provided written consent to participate after receiving information about the study. This study was conducted in strict compliance with the principles of the Declaration of Helsinki.

### 2.2. Study design

This was an observational, cross-sectional, analytical-descriptive, and quantitative study reported in accordance with the Strengthening the Reporting of Observational Studies in Epidemiology guidelines.

### 2.3. Study participants

The diagnostic criteria for long COVID included a previous diagnosis of COVID-19, presentation of symptoms at least 4 weeks after the acute phase of the disease, and the presence of symptoms for at least 3 months, with symptoms appearing no later than 2 months after infection. COVID-19 was diagnosed using positive polymerase chain reaction tests and was characterized by fatigue, shortness of breath, cough, joint pain, chest pain, muscle pain, headache, and other symptoms that cannot be attributed to any other cause (4).

**Figure 1:**
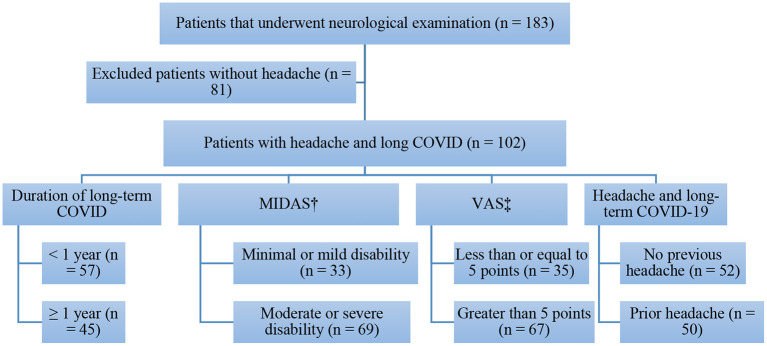
Flowchart of patients who had headache with long-term COVID-19 in the present study. Source: Authors themselves, 2020–2021. ^†^Migraine Disability Assessment. ^‡^Visual Analog Pain Scale.

From August 2020 to November 2021, 183 patients underwent neurological evaluation, 81 (44.26%) of whom were excluded for not having headaches associated with long COVID. Thus, the final sample consisted of 102 patients with long COVID symptoms. The patients were divided into different groups based on the following factors: (i) prior history of headache: with a prior history of headache (*n* = 50) and without a prior history of headache (*n* = 52); (ii) duration of long COVID: up to 12 months (*n* = 57) and 12 months or longer (*n* = 45); (iii) migraine disability assessment (MIDAS) questionnaire results: minimal or mild disability (*n* = 33) and moderate or severe disability (*n* = 69); (iv) visual analog scale for pain (VAS) score: ≤5 (*n* = 35) and >5 (*n* = 67); (v) for patients with a prior history of headache (*n* = 50): characteristics of headache before and after contracting COVID-19 (Figure 1).

### 2.4. Procedure

An interview was conducted to collect sociodemographic data, general clinical data, previous headache data (pain characteristics, frequency, type, headache relief with analgesia, and associated symptoms), and current headache data (pain characteristics, frequency, type, duration, periodicity, headache relief with analgesia, headache worsening after contracting COVID-19, and associated symptoms). In addition, the MIDAS questionnaire (15) was used to assess the impact of pain on the quality of life and degree of disability (minimal, mild, moderate, or severe disability) of patients, and the VAS (16) was used to determine their intensity of headache using a scale from zero (no pain) to 10 (severe pain).

### 2.5. Statistical analysis

The collected data were analyzed using GraphPad Prism™ version 5.0 software (GraphPad Software, Inc., San Diego, CA, USA). To assess the distribution of epidemiological characteristics of the collected data, simple descriptive and inferential statistical methods were applied. Categorical variables were analyzed using Fisher's exact test and reported as the frequency and percentage. The association between the individual exposure factors of patients and clinical outcome (headache associated with long COVID) was evaluated using logistic regression, from which the raw odds ratio (for each exposure factor) and respective confidence interval values were calculated. An α level of 0.05 was adopted to reject the null hypothesis.

## 3. Results

Long COVID headache was observed most frequently in women aged more than 39 years and in individuals with more than 9 years of education. The average duration of long COVID in the patients was 321.46 days; more than half of the patients did not have a history of headaches prior to contracting COVID-19, and those with a prior history of headache reported experiencing worsening pain in the long COVID phase. The characteristics of long COVID headache consisted of the following: a “pressure” or “squeezing” headache, frequency between two and five times each week, duration more than 6 h, and pain in the bilateral temporal cranial region. Additionally, the major associated symptoms were photophobia and phonophobia (Table 1).

**Table 1:** Characteristics of the patients with headache and long COVID-19 in this study.

**Variables**	**Total**
**Sex (** ***n*** **, %)**	
Male	19 (18.63%)
Female	83 (81.37%)
**Age (years)**	
≤39 years	38 (37.25%)
>39 years	64 (62.75%)
**Education (** ***n*** **, %)**	
≤9 years	4 (3.92%)
>9 years	98 (96.08%)
**Average duration of long COVID-19 (days)**	321.46
**Presence of headache before COVID-19 (** ***n*** **, %)**	
Yes	50 (49.02%)
No	52 (50.98%)
**Presence of exacerbated headache after COVID-19 (** ***n*** **, %)**	
Yes	43 (86.00%)
No	7 (14.00%)
**Headache characteristic after COVID-19 (** ***n*** **, %)**	
Throbbing	28 (27.45%)
Pressing	59 (57.84%)
Burning	7 (6.86%)
Cluster	8 (7.84%)
**Headache frequency after COVID-19 (** ***n*** **, %)**	
Daily	28 (27.45%)
2–5 times per week	45 (44.12%)
Weekly	19 (18.63%)
Monthly	10 (9.80%)
**Headache duration after COVID-19 (** ***n*** **, %)**	
≤6 h	50 (49.02%)
>6 h	52 (50.98%)
**Headache periodicity after COVID-19 (** ***n*** **, %)**	
< 2 periods of the day	55 (53.92%)
≥2 periods of the day	47 (46.08%)
**Headache location after COVID-19 (** ***n*** **, %)**	
Diffuse	43 (18.94%)
Frontal	48 (21.15%)
Bi-temporal	51 (22.47%)
Unilateral	32 (14.10%)
Occipital	53 (23.35%)
**Headache after COVID-19 (** ***n*** **, %)**	
Improvement without medication	18 (17.65%)
Partial improvement with analgesic use	18 (17.65%)
Total improvement with analgesic use	61 (59.80%)
No improvement with analgesic use	5 (4.90%)
**Symptoms associated with post-COVID-19 headache (** ***n*** **, %)**	
Dizziness and/or vertigo	52 (22.22%)
Photophobia	56 (23.93%)
Phonophobia	54 (23.08%)
Osmophobia	37 (15.81%)
Nausea and/or vomiting	35 (14.96%)
No symptoms associated	23 (22.55%)
**Number of associated symptoms (** ***n*** **, %)**	
≤2 symptoms	30 (37.97%)
>2 symptoms	49 (62.03%)
**Presence of comorbidities (** ***n*** **, %)**	
Systemic arterial hypertension	27 (26.47%)
Diabetes	6 (5.88%)

Belém-PA, 2020–2021.

Source: Research protocol.

**Figure 2:**
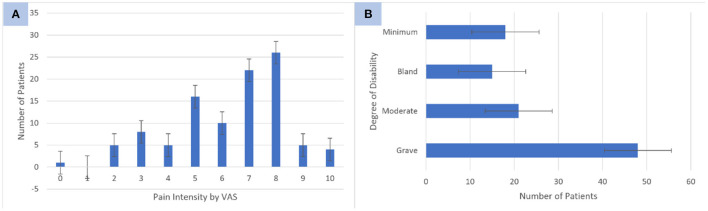
**(A)** Pain intensity according to Visual Analog Pain Scale (VAS) scores in patients with headache and long-term COVID-19. **(B)** Degree of disability of patients with headache and long-term COVID-19. Source: research protocol. Standard error ≤ 0.05.

Headaches with an intensity of higher than five points on the VAS were observed in 65.68% of the patients (67/102), and the highest reported VAS score was eight points (25.49%). Severe disability was observed in 47.05% of the patients (Figures 2A, B).

### 3.1. Prior headache vs. new-onset headache associated with long COVID

The pain characteristics before and after contracting COVID-19 in patients with a prior history of headache are presented in Table 2. Before contracting COVID-19, most of the patients reported total improvement in the condition by using analgesics (*p* = 0.005). However, in the long COVID phase, they experienced the following: an increased frequency of pain both daily (*p* = 0.022) and two to five times each week (*p* = 0.012), and only partial improvement in the pain condition after taking medications (*p* = 0.002).

**Table 2:** Comparison of pain characteristics in patients with a history of headache before and after COVID-19 in this study.

**Variables**	**COVID-19**		***p*-value**
	**Before (*****n*** = **50)**	**After (*****n*** = **50)**	
**Characteristic (** ***n*** **, %)**			
Throbbing	21 (42.00%)	14 (28.00%)	0.208
Pressing	23 (46.00%)	28 (56.00%)	0.423
Burning	2 (4.00%)	2 (4.00%)	1.000
Cluster	4 (8.00%)	6 (12.00%)	0.740
**Frequency (** ***n*** **, %)**			
Daily	3 (6.00%)	12 (24.00%)	**0.022**
2–5 times per week	12 (24.00%)	25 (50.00%)	**0.012**
Weekly	14 (28.00%)	9 (18.00%)	0.247
Monthly	21 (42.00%)	4 (8.00%)	**0.000**
**Location (** ***n*** **, %)**			
Diffuse	16 (16.33%)	25 (21.93%)	0.383
Frontal	22 (22.45%)	22 (19.30%)	0.612
Bi-temporal	30 (30.61%)	27 (23.68%)	0.279
Unilateral	14 (14.29%)	15 (13.16%)	0.843
Occipital	16 (16.33%)	25 (21.93%)	0.383
**Improvement of pain (** ***n*** **, %)**			
Improvement without medication	10 (20.00%)	6 (12.00%)	0.413
Partial improvement with analgesic use	19 (38.00%)	35 (70.00%)	**0.002**
Total improvement with analgesic use	19 (38.00%)	6 (12.00%)	**0.005**
No improvement with analgesic use	2 (4.00%)	3 (6.00%)	1.000
**Associated symptoms (** ***n*** **, %)**			
No	17 (34.00%)	7 (14.00%)	**0.033**
Yes	33 (66.00%)	43 (86.00%)	
**Types of associated symptoms (** ***n*** **, %)**			
Dizziness and/or vertigo	14 (16.28%)	29 (22.48%)	0.130
Photophobia	23 (26.74%)	33 (25.58%)	0.759
Phonophobia	18 (20.93%)	30 (23.26%)	0.418
Osmophobia	14 (16.28%)	20 (15.50%)	0.853
Nausea and/or vomiting	17 (19.77%)	17 (13.18%)	0.455
**Number of associated symptoms (** ***n*** **, %)**			
≤2 symptoms	17 (51.52%)	15 (34.88%)	0.166
>2 symptoms	16 (48.48%)	28 (65.12%)	

Belém-PA, 2020–2021.

Fisher's exact test.

Bold value indicates that these comparisons were statistically significant.

### 3.2. Headache associated within long COVID

Patients who had a history of headache prior to contracting COVID-19 reported having a longer duration of long COVID headache (*p* = 0.031) and only partial improvement in pain with medications (*p* = 0.045) compared to those without previous headache. The other variables for long COVID headache demonstrated no differences between patients with previous headache and those without (Table 3).

**Table 3:** Characteristics of patients with headache and long COVID-19 stratified by MIDAS^†^ grades, long COVID-19 durations, and VAS^‡^ scores.

**Variable**				**MIDAS** ^**†**^			**VAS**^**‡**^ **scores**			**Long COVID-19 duration**		
	**Patients without prior headache (*****n*** = **52)**	**Patient with previous headache (*****n*** = **50)**	***p*** **-value**	**Minimal or mild disability (*****n*** = **33)**	**Moderate or severe disability (*****n*** = **69)**	***p*** **-value**	≤ **5 points (*****n*** = **35)**	>**5 points (*****n*** = **67)**	***p*** **-value**	<**1 year (*****n*** = **57)**	≥**1 year (*****n*** = **45)**	***p*** **-value**
**Sex (** ***n*** **, %)**												
Male	12 (23.08%)	7 (14.00%)	0.311	10 (30.30%)	9 (13.04%)	0.055	8 (22.86%)	11 (16.42%)	0.434	7 (12.28%)	12 (26.67%)	0.076
Female	40 (76.92%)	43 (86.00%)		23 (69.70%)	60 (86.96%)		27 (77.14%)	56 (83.58%)		50 (87.72%)	33 (73.33%)	
**Age (** ***n*** **, %)**												
≤39 years	17 (32.69%)	21 (42.00%)	0.413	10 (30.30%)	28 (40.58%)	0.384	11 (31.43%)	27 (40.30%)	0.399	25 (43.86%)	13 (28.89%)	0.150
>39 years	35 (67.31%)	29 (58.00%)		23 (69.70%)	41 (59.42%)		24 (68.57%)	40 (59.70%)		32 (56.14%)	32 (71.11%)	
**Characteristic (** ***n*** **, %)**												
Throbbing	14 (26.92%)	14 (28.00%)	1.000	6 (18.18%)	22 (31.88%)	0.164	9 (25.71%)	19 (28.36%)	0.819	13 (22.81%)	15 (33.33%)	0.269
Pressing	31 (59.62%)	28 (56.00%)	0.841	24 (72.73%)	35 (50.72%)	0.053	22 (62.86%)	37 (55.22%)	0.529	34 (59.65%)	25 (55.56%)	0.691
Burning	5 (9.62%)	2 (4.00%)	0.437	1 (3.03%)	6 (8.70%)	0.423	2 (5.71%)	5 (7.46%)	1.000	5 (8.77%)	2 (4.44%)	0.460
Cluster	2 (3.85%)	6 (12.00%)	0.156	2 (6.06%)	6 (8.70%)	0.720	2 (5.71%)	6 (8.96%)	0.711	5 (8.77%)	3 (6.67%)	0.731
**Frequency (** ***n*** **, %)**												
Daily	16 (30.77%)	12 (24.00%)	0.509	3 (9.09%)	25 (36.23%)	**0.004**	6 (17.14%)	22 (32.84%)	0.106	19 (33.33%)	9 (20.00%)	0.180
2–5 times per week	20 (38.46%)	25 (50.00%)	0.318	17 (51.52%)	28 (40.58%)	0.394	16 (45.71%)	29 (43.28%)	0.836	21 (36.84%)	24 (53.33%)	0.111
Weekly	10 (19.23%)	9 (18.00%)	1.000	8 (24.24%)	11 (15.94%)	0.415	7 (20.00%)	12 (17.91%)	0.794	9 (15.79%)	10 (22.22%)	0.450
Monthly	6 (11.54%)	4 (8.00%)	0.741	5 (15.15%)	5 (7.25%)	0.286	6 (17.14%)	4 (5.97%)	0.087	8 (14.04%)	2 (4.44%)	0.178
**Duration (** ***n*** **, %)**												
≤6 h	31 (59.62%)	19 (38.00%)	**0.031**	25 (75.76%)	25 (36.23%)	**0.000**	25 (71.43%)	25 (37.31%)	**0.001**	31 (54.39%)	19 (42.22%)	0.238
>6 h	21 (40.38%)	31 (62.00)		8 (24.24%)	44 (63.77%)		10 (28.57%)	42 (62.69%)		26 (45.61%)	26 (57.78%)	
**Periodicity (** ***n*** **, %)**												
<2 periods of the day	29 (55.77%)	26 (52.00%)	0.842	20 (60.61%)	35 (50.72%)	0.399	22 (62.86%)	33 (49.25%)	0.214	36 (63.16%)	19 (42.22%)	**0.045**
≥2 periods of the day	23 (44.23%)	24 (48.00%)		13 (39.39%)	34 (49.28%)		13 (37.14%)	34 (50.75%)		21 (36.84%)	26 (57.78%)	
**Location (** ***n*** **, %)**												
Diffuse	18 (15.93%)	25 (21.93%)	0.309	12 (16.90%)	31 (19.87%)	0.715	7 (10.77%)	36 (22.22%)	0.060	22 (19.82%)	21 (18.10%)	0.865
Frontal	26 (23.01%)	22 (19.30%)	0.519	14 (19.72%)	34 (21.79%)	0.861	16 (24.62%)	32 (19.75%)	0.472	24 (21.62%)	24 (20.69%)	0.872
Bi-temporal	24 (21.24%)	27 (23.68%)	0.750	18 (25.35%)	33 (21.15%)	0.496	17 (26.15%)	34 (20.99%)	0.481	25 (22.52%)	26 (22.41%)	1.000
Unilateral	17 (15.04%)	15 (13.16%)	0.707	11 (15.49%)	21 (13.46%)	0.684	10 (15.38%)	22 (13.58%)	0.833	14 (12.61%)	18 (15.52%)	0.571
Occipital	28 (24.78%)	25 (21.93%)	0.640	16 (22.54%)	37 (23.72%)	0.867	15 (23.08%)	38 (23.46%)	1.000	26 (23.42%)	27 (23.28%)	1.000
**Associated symptoms (** ***n*** **, %)**												
No	16 (30.77%)	7 (14.00%)	0.058	10 (30.33%)	13 (18.84%)	0.213	15 (42.86%)	8 (11.94%)	**0.000**	16 (28.07%)	7 (15.56%)	0.157
Yes	36 (69.23%)	43 (86.00%)		20 (66.67%)	56 (81.15%)		20 (57.14%)	59 (88.05%)		41 (71.92%)	38 (84.44%)	
**Type of associated symptoms (** ***n*** **, %)**												
Dizziness and/or vertigo	23 (21.90%)	29 (22.48%)	1.000	11 (22.00%)	41 (22.28%)	1.000	8 (16.67%)	44 (23.66%)	0.336	29 (25.44%)	23 (19.17%)	0.344
Photophobia	23 (21.90%)	33 (25.58%)	0.541	15 (30.00%)	41 (22.28%)	0.266	12 (25.00%)	44 (23.66%)	0.850	26 (22.81%)	30 (25.00%)	0.649
Phonophobia	24 (22.86%)	30 (23.26%)	1.000	11 (22.00%)	43 (23.37%)	1.000	11 (22.92%)	43 (23.12%)	1.000	24 (21.05%)	30 (25.00%)	0.442
Osmophobia	17 (16.19%)	20 (15.50%)	1.000	7 (14.00%)	30 (16.30%)	0.828	10 (20.83%)	27 (14.52%)	0.373	18 (15.79%)	19 (15.83%)	1.000
Nausea and/or vomiting	18 (17.14%)	17 (13.18%)	0.462	6 (12.00%)	29 (15.76%)	0.828	7 (14.58%)	28 (15.05%)	1.000	17 (14.91%)	18 (15.00%)	1.000
**Number of associated symptoms (** ***n*** **, %)**												
≤2 symptoms	15 (41.67%)	15 (34.88%)	0.642	15 (45.45%)	15 (26.79%)	0.103	11 (31.43%)	19 (32.20%)	1.000	18 (31.58%)	12 (31.58%)	1.000
>2 symptoms	21 (58.33%)	28 (65.12%)		18 (54.55%)	41 (73.21%)		24 (68.57%)	40 (67.80%)		39 (68.42%)	26 (68.42%)	
**Improvement of pain (** ***n*** **, %)**												
Improvement without medication	12 (23.08%)	6 (12.00%)	0.195	8 (24.24%)	10 (14.49%)	0.270	13 (37.14%)	5 (7.46%)	**0.000**	15 (26.32%)	3 (6.67%)	**0.010**
Total improvement with analgesic use	26 (50.00%)	35 (70.00%)	**0.045**	16 (48.48%)	45 (65.22%)	0.132	16 (45.71%)	45 (67.16%)	0.054	31 (54.39%)	30 (66.67%)	0.228
Partial improvement with analgesic use	12 (23.08%)	6 (12.00%)	0.195	8 (24.24%)	10 (14.49%)	0.270	4 (11.43%)	14 (20.90%)	0.283	7 (12.28%)	11 (24.44%)	0.124
No improvement with analgesic use	2 (3.85%)	3 (6.00%)	0.675	1 (3.03%)	4 (5.80%)	0.667	2 (5.71%)	3 (4.48%)	0.996	4 (7.02%)	1 (2.22%)	0.380
**Visual Analog Pain Scale (VAS;** ***n*****, %)**												
≤5 points	20 (38.46%)	15 (30.00%)	0.409	17 (51.52%)	18 (26.09%)	**0.014**	-	-	-	22 (38.60%)	13 (28.89%)	0.401
>5 points	32 (61.54%)	35 (70.00%)		16 (48.48%)	51 (73.91%)		-	-		35 (61.40%)	32 (71.11%)	
**Migraine Disability Assessment (MIDAS;** ***n*****, %)**												
Inability minimum or bland	18 (34.62%)	15 (30.00%)	0.675	-	-	-	17 (48.57%)	16 (23.88%)	**0.014**	17 (29.82%)	16 (35.56%)	0.670
Inability moderate or grave	34 (65.38%)	35 (70.00%)		-	-		18 (51.43%)	51 (76.12%)		40 (70.18%)	29 (64.44%)	

Belém/PA, 2020–2021.

Source: Research protocol.

Fisher's exact test.

^†^Migraine Disability Assessment.

^‡^Visual Analog Scale of Pain.

Bold value indicates that these comparisons were statistically significant.

On comparing the disability generated by headache in the patients with long COVID, it was observed that patients with moderate or severe disability experienced daily headaches more frequently (*p* = 0.004) and a greater intensity of pain, as assessed by the VAS (*p* = 0.014), than patients with minimal or mild disability. Patients with minimal or mild disability had a shorter pain duration than those with moderate or severe disability (*p* = 0.000; Table 3).

Most of the patients with long COVID with VAS scores more than five points reported a long duration of pain (*p* = 0.001), and only 7.41% of them reported an improvement in pain without the use of medications (*p* = 0.000). This group of patients also presented with symptoms associated with headache (*p* = 0.000) and moderate or severe disability according to the MIDAS (*p* = 0.014; Table 3).

Individuals with a duration of long COVID duration of more than 1 year had headaches less than twice a day (*p* = 0.045) and exhibited an improvement in pain without the use of medications (*p* = 0.010) compared to patients with a long COVID duration of more than or equal to 1 year (Table 3).

### 3.3. Multiple logistic regression

Hospitalization, symptoms of back pain, insomnia, and paraesthesia were the factors that were demonstrated to be associated with headache in patients with long COVID (Table 4).

**Table 4:** Multiple logistic regression of factors that may contribute to headache in long COVID-19.

**Variables**	**Regression coefficient**	***p*-value**	**Odds ratio**	**95% CI**
Hospital internment	1.1014	**0.0325**	3.0082	1.10–8.26
Back pain	1.3867	**0.0316**	4.0017	1.13–14.17
Insomnia	1.1423	**0.0059**	3.1339	1.39–7.06
Paraesthesia	1.0152	**0.0165**	2.7600	1.20–6.33
Sex	−0.2221	0.6066	0.8009	0.34–1.86
Age	0.0116	0.4287	1.0116	0.98–1.04
Schooling	0.1647	0.3006	1.1790	0.89–1.61
Income	−0.3010	0.4088	0.7401	0.36–1.51
Long COVID-19 duration	0.0015	0.4780	1.0015	1.00–1.01
Tiredness	0.6501	0.0926	1.9157	0.90–4.09
Weakness	−0.0552	0.9197	0.9463	0.32–2.76
Dyspnoea	−0.3987	0.4254	0.6712	0.25–1.79
Myalgia	0.6404	0.2120	1.8973	0.69–5.19
Pain: thoracic	−0.1139	0.8689	0.8923	0.23–3.45
Alopecia	0.0470	0.9190	1.0482	0.42–2.60
Depression symptoms	0.5894	0.3726	1.8029	0.49–6.59
Anxiety symptoms	0.5044	0.2268	1.6559	0.73–3.75
Anosmia	−0.6151	0.1597	0.5406	0.23–1.27
Ageusia	−0.2923	0.4845	0.7466	0.33–1.69
Light cognitive decline	0.5573	0.1147	1.7460	0.87–3.49

Belém/PA, 2020–2021.

Source: Research protocol.

Bold value indicates that these comparisons were statistically significant.

## 4. Discussion

After developing long COVID, the patients in this study experienced headaches with different characteristics than those with headaches prior to contracting COVID-19. Additionally, patients with a higher degree of disability according to the MIDAS presented with a higher severity of long COVID, with a chronification tendency and longer duration of the pain condition. They also reported associated symptoms of photophobia, nausea, and/or vomiting and a reduced response to analgesic therapy. Patients with a duration of long COVID >1 year reported having headaches at least twice a day, and <7% of this group of patients demonstrated an improvement in pain without the use of analgesic medications. Therefore, some factors, such as hospitalization, back pain, insomnia, and paraesthesia, seem to be associated with headaches in patients with long COVID.

Headaches in patients with ID were present in those with or without a previous history of headache; however, most patients with a history of headache prior to COVID-19 reported worsening of the condition (3, 6, 7, 10), including the participants in this study. Al-Hashel et al. (17) conducted a cross-sectional study in Kuwait on 121 patients with headache associated with long COVID and observed that 64.5% of participants reported prior migraines, 9.1% reported having a history of tension-type headaches, and 26.4% reported that their headaches began after becoming infected with COVID-19. Patients with a history of headache reported increased numbers of days with pain, those with migraine increased their use of abortive headache medications, and those with tension-type headache exhibited a significant increase in the severity and frequency of pain (17).

The phenotype of headache in Long COVID varies. Many people may experience pain with tension-type headache characteristics (pressing pain that is frontal or bilateral and that does not worsen with physical activity), while other patients may experience pain with migraine characteristics (pulsatile pain that is unilateral, worsened by physical activity, and that may or may not be accompanied by phonophobia, photophobia, nausea, or vomiting, and worsening with physical activities) (10). Our In this study, participants reported long COVID headaches with “pressing” or “squeezing” characteristics. Similar results were reported in two studies from Spain, in which 580 (18) and 112 individuals (19) with long COVID reported a higher prevalence of headaches with “pressing” or “squeezing” characteristics, respectively, as opposed to headaches with “pulsating” or “stabbing” patterns of pain, which is similar to what occurs with tension-type headaches (6).

Similar results to those found in our study were reported by a cross-sectional study of 201 individuals in the city of Madrid in which no difference in the prevalence rates of headache was found between patients with long COVID with migraine prior to contracting COVID-19 and those without migraine prior to contracting COVID-19 (20). Thus, it is possible that patients with or without headaches prior to contracting COVID-19 experience similar types of pain in the long COVID phase, indicating that a certain type of headache may be specific and inherent to long COVID.

**Figure 3:**
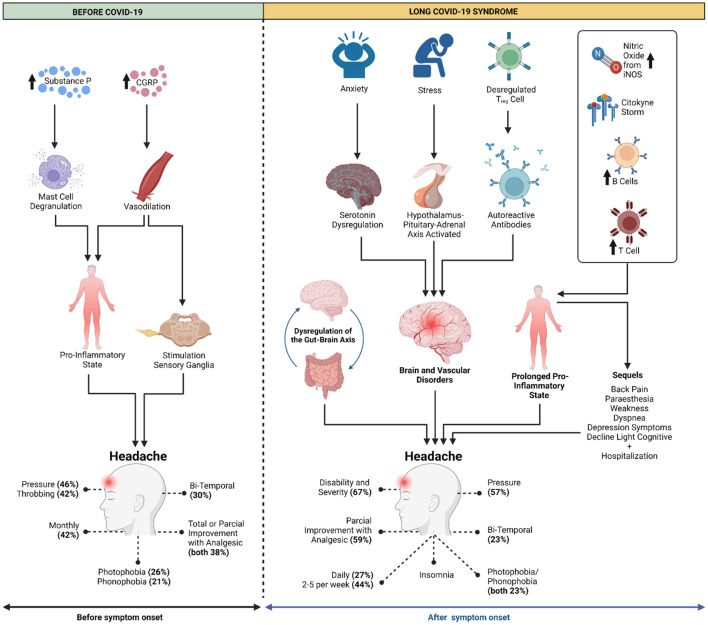
Headache prior to COVID-19 is explained by the release of substance P and calcitonin gene related peptide (CGRP) to induce mast cell degranulation and vasodilation, respectively, leading to a proinflammatory state that, when associated with stimulation of sensory ganglia, trigger headache. In long COVID-19 syndrome, stressors and anxiety could lead to changes in neurotransmitters and neurohormonal pathways, triggering brain and vascular changes. In addition, fragments of remaining pathogens may induce the creation of autoantibodies and increase the release of inflammatory cells, such as cytokines and B and T cells, which are associated with the increased production of nitric oxide by inducible nitric oxide synthetase (iNOS). This could lead to a prolonged proinflammatory state, eventually leading to the main post-COVID-19 sequelae. Moreover, disorders of the gut flora can alter the gut-brain axis. All of these factors could contribute to new cases of post-COVID-19 headache and/or worsening of pain in patients with previous headache. Created with BioRender.

Tension-type headaches may be related to an individual's state of stress (21). Stressors and anxiety, which were highly prevalent complaints during the COVID-19 pandemic, may be related to the initiation and/or worsening of pain in patients with headache in long COVID, as it can cause the dysregulation of neurotransmitters, such as serotonin, and cause imbalances in the hypothalamic–pituitary–adrenal axis. Therefore, it is likely that fragments of pathogens remaining from the acute infection period can disrupt regulatory T cells, inducing autoantibody formation and causing cerebral and vascular damage (22) (Figure 3).

Most of the participants in this study were women aged >39 years. Corroborating our findings, a previous study involving 121 patients demonstrated that 84% of patients who reported headache during long COVID syndrome were women with a mean age of 35 years (17). In addition, Caronna et al. (6) observed that most patients with long COVID headaches were aged >45 years. Furthermore, a meta-analysis comprising >100 studies revealed that the prevalence of chronic headache increases with age (23).

Phonophobia and photophobia were the associated symptoms that were frequently reported by the participants of this study. Similar associated symptoms were reported by 112 health professionals with headache and COVID-19 (19), as well as dizziness and vertigo (24). In addition, other studies have also reported that patients with long COVID headache presented with phonophobia and photophobia as associated symptoms (7, 25). Moreover, a similar study reported that the same symptoms were associated with tension-type headache (26), which may reinforce the similarities among tension-type headache and long COVID headache.

An association between the intensity and degree of disability related to long COVID headache was observed in the participants of this study. According to López et al. (18), 64% (83/130) of hospitalized patients with COVID-19 had severe headaches, and more than half of the interviewees exhibited a moderate-to-severe degree of disability. A study involving 2,194 patients with COVID-19 reported headache with a median intensity of 7 on a scale from 0 to 10 (27). A case-controlled study developed in the Egypt on long COVID headache reported that patients with a previous diagnosis of migraine evaluated their actual pain with a median of 5.5 points on the VAS. Meanwhile, patients with headache attributed to viral system infection reported a median of two points on the VAS, and patients with NDPH reported median VAS scores of five points (28). Although no data were found in the literature regarding the correlation between pain intensity and disability in long COVID, it is possible that patients with severe pain have a high degree of disability.

The present study found that patients with long COVID experienced headaches with long durations. Studies on the acute period of COVID-19 reported that the duration of pain of headache can vary from hours to days or that it can persist for more than 30 days (27, 29). Similarly, long COVID headache presents similar characteristics. A multicentric study from Spain reported that participants experienced pain with durations varying between 14 days to >9 months, and their univariate analysis found that age, female sex, pain intensity, headaches with “pressing” or “throbbing” characteristics, photophobia, and phonophobia were associated a long duration of headache in long COVID (7). Although no data were found in the literature regarding the correlation between the duration of pain and disability of headache in long COVID, it is possible that a longer duration of pain contributes to a more-disabling headache.

Our results indicate an association between headache and insomnia. According to Cho et al. (30), patients with tension-type headaches presented with more sleep disturbances than patients without headaches. Furthermore, a systematic review described the correlation between migraine and chronic sleep problems (31). It is speculated that brain areas, such as the hypothalamus; neurotransmitters, such as serotonin and dopamine; and the glymphatic system are common pathophysiological pathways between headache and insomnia (32). This explains the possible association between long COVID headache and insomnia.

Although the pathophysiology of long COVID headache is yet to be fully elucidated, it is likely that immunological alterations, cytokine storms, and the proinflammatory state of long COVID may trigger a dysautonomic syndrome among other symptoms, such as dizziness (33). It is believed that nitric oxide (NO), among other factors, plays a role in the pathogenesis of tension-type headache to mediate vasodilation, which causes pain (34). An increase in the production of NO from inducible nitric oxide synthase (iNOS) has been observed as a response to COVID-19 (35). It is likely that, in addition to the prolonged proinflammatory state of patients with long COVID (3), these patients also exhibit an increase in the production of NO by iNOS, which could contribute to the onset of headaches in these patients. In addition, increases in B cell and T cell proliferation associated with a cytokine storm could lead to a prolonged proinflammatory state (22), which likely contributes to headache in patients with long COVID as well as to the manifestation of long COVID sequelae, which may also be related to headache at this stage of the disease.

Mechanisms that elicit vasodilation and a proinflammatory state may be related to the pathophysiology of long COVID. The connection between SARS-CoV-2 and angiotensin-converting enzyme (2) leads to an imbalance between the pro- and anti-inflammatory pathways, additionally leading to overactivation of the renin–angiotensin system. Overactivated angiotensin receptor (AT1R) results in cytokine storm syndrome and overproduction of proinflammatory cytokines, which can lead to vasoconstriction, inflammation, oxidative stress, and neurological dysfunction (36).

Tryptophan metabolism is altered in patients with long COVID. During COVID-19 infection, it is possible that there is an increase in the levels of kynurenine, whose function is the enzymatic degradation of tryptophan (37). An imbalance between tryptophan and kynurenine levels can trigger a headache attack (38). Therefore, it is possible that this mechanism may be associated with the symptoms of long COVID.

Among the limitations of this study, it should be noted that prior headaches were evaluated according to retrospective reports of patients, which could introduce a memory bias. However, headache is a common disease, and its carriers are, in most cases, clear about their individual characteristics. The MIDAS is a questionnaire designed to assess migraine, the use of which could introduce instrumentation bias; however, we used it to assess other headaches due to the absence of another validated instrument that was equally capable of measuring the disability brought about by headaches.

In this study, we investigated headache symptoms in patients with long COVID for up to 15 months, and this is the first study in the Brazilian Amazon demonstrating the chronicity of headache in long COVID. However, further studies that follow up with patients with COVID-19 for a period longer than that in the present study (15 months) should be conducted. Additionally, cohort studies assessing and reassessing the signs and symptoms of long COVID headache to define the changes in the clinical findings of headache and disability over time in long COVID are needed, as this still represents an important gap in knowledge to be filled.

In conclusion, long COVID headache is a disabling and intense manifestation that persists for months after the acute period of COVID-19, exhibiting a chronification tendency. Patients with a history of headache report a greater exacerbation of pain and, sometimes, different pain characteristics between headaches occurring during the long COVID phase and headaches occurring prior to this phase. Notably, hospitalization, paraesthesia, back pain, and insomnia were associated with headaches in patients with long COVID.

The results of the present study can be explained by the involvement of multiple organs and systems, both before and during COVID-19 infection, as this can directly or indirectly alter the central nervous system and cause headache. This study is the first in the Brazilian Amazon to describe headache associated with long COVID and its long-term involvement in the exacerbation of symptoms. Although the results of the present research are important, we suggest performing further prospective studies to assess and monitor the signs and symptoms of headache in patients with long COVID, in addition to studies involving pain therapy, as this is still an important gap in scientific knowledge in this field.

## Data availability statement

The raw data supporting the conclusions of this article will be made available by the authors, without undue reservation.

## Ethics statement

The studies involving human participants were reviewed and approved by Ethical Committee in Research of State University of Pará. The patients/participants provided their written informed consent to participate in this study.

## Author contributions

AR and LF: conceptualization, investigation, figures, writing—original draft, and writing—review and editing. TB, BB, CS, and NS: writing—original draft and writing—review and editing. AP, AD, and ES: writing—review and editing. JQ and LF: supervision and project administration. All authors contributed to the article and approved the submitted version.
